# Molecular characterization, chemical profile and biological properties of essential oils from *Chamaemelum nobile* (L.) flowers of Morocco: in vitro and *in silico* studies

**DOI:** 10.3389/fchem.2025.1539872

**Published:** 2025-02-04

**Authors:** El-Mehdi El-Assri, Youssef El-Assri, Rajae El Brahimi, Mohamed El fadili, Asmae Baghouz, Hatem A. Abuelizz, Sara Er-Rahmani, Amal Lahkimi, Abdelhak Bouia

**Affiliations:** ^1^ Laboratory of Biotechnology, Environment, Agri-Food and Health, Faculty of Sciences Dhar El Mahraz, Sidi Mohamed Ben Abdellah University, Fez, Morocco; ^2^ Laboratory of Engineering, Electrochemistry, Modeling and Environment, Faculty of Sciences Dhar El Mahraz, Sidi Mohamed Ben Abdellah University, Fez, Morocco; ^3^ LIMAS Laboratory, Department of chemistry, Faculty of Sciences Dhar El Mahraz, Sidi Mohamed Ben Abdellah University, Fez, Morocco; ^4^ Laboratory of Biotechnology, Conservation and Valorisation of Naturals Resources, Faculty of Sciences Dhar El Mehraz, Sidi Mohamed Ben Abdellah University, Fez, Morocco; ^5^ Department of Pharmaceutical Chemistry, College of Pharmacy, King Saud University, Riyadh, Saudi Arabia; ^6^ Department of Chemistry, Nis Interdepartmental Centre, University of Turin, Turin, Italy

**Keywords:** antimicrobial activity, antioxidant activity, *Chamaemelum nobile* (L.), essential oil, GC-MS, in silico studies

## Abstract

**Introduction:**

This study investigated the antioxidant, antimicrobial, and insecticidal properties of *Chamaemelum nobile* (L.) essential oil (CN-EO), harvested in Taounate, Morocco. The molecular composition and chemical profile of CN-EO were also characterized.

**Methods:**

The CN-EO was extracted using a Clevenger apparatus. Its chemical composition was analyzed using gas chromatography-mass spectrometry (GC-MS). Antioxidant activity was evaluated using the DPPH assay, while antimicrobial properties were assessed via the disk diffusion method to measure inhibition zones against various bacterial and fungal strains. Insecticidal activity was tested through bioassays to determine insect mortality and repellency rates. Phylogenetic analysis of DNA sequences was conducted to confirm the species identity.

**Results:**

GC-MS analysis identified 24 compounds in CN-EO, with β-Oplopenone (18.66%), Spathulenol (14.90%), and Himachalene (12.47%) as major constituents. CN-EO exhibited strong antioxidant activity (IC_50_ = 135.8 ± 1.03 μg/mL). Antimicrobial assays revealed inhibition zones of up to 20.67 ± 0.58 mm (*Staphylococcus aureus*) and antifungal inhibition of 40.42% ± 2.82% against Aspergillus flavus. Insecticidal tests showed total insect mortality at 166 µL/L within 48 h and a 60% repellent effect. Phylogenetic analysis of the DNA sequence revealed a 99.22% similarity with *Chamaemelum nobile* (L.).

**Conclusion:**

These results demonstrate the significant potential of Moroccan CN-EO in phytomedicine. It exhibits a wide range of biological activities and shows great promise as a natural antioxidant, antimicrobial agent, antifungal, and insecticide.

## 1 Introduction

Throughout history, civilizations have used aromatic and medicinal plants to protect their crops from pests. However, in the 20th century, synthetic chemical insecticides became widely adopted. While these chemicals effectively safeguard crops, they present considerable risks because of their negative impacts on the environment and human health. They persist and accumulate in ecosystems and the human body, often leading to chronic illnesses and other health issues (Jallow et al., 2017; Allali et al., 2022). This growing concern has highlighted the importance of developing alternative solutions that are environmentally sustainable and promote human health. *Callosobruchus maculatus*, commonly known as the “pea weevil,” belongs to the Chrysomelidae family and is recognized for infesting chickpeas (*Cicer arietinum*) and other legumes, making it a significant pest in stored grains. Its detrimental effects are especially significant in Africa, as well as in tropical regions and South America (Ogunkanmi et al., 2018; Bidar et al., 2021). The larvae of the pea weevil cause damage to the grains by feeding on them after hatching, resulting in reduced seed weight, diminished nutritional value, and compromised germination capacity (Osa and Georgina, 2016).

In this context, plant essential oils (EOs) are a diverse group of lipophilic, biologically active, and volatile secondary compounds. They are essential for protecting plants against various threats, particularly fungi, insects, and pathogenic bacteria. Essential oils have been extensively researched globally for their biocidal attributes, especially in post-harvest plant preservation. Research has shown their effectiveness against pests of cereal, legume, and citrus crops, as well as against various microorganisms present in foodstuffs (Atif et al., 2020; Kavallieratos et al., 2020).

*C. nobile* (L.), widely known as Roman chamomile, is a perennial herbaceous plant characterized by a branched rhizome and numerous stems, each bearing small, enduring leaves. Together, they create a sprawling mat of aromatic foliage. During the summer and early autumn, it produces flowers with white rays and yellow centres, similar to daisy blossoms (Dezfooli et al., 2012; El-Assri et al., 2021a). Originally from southern Europe, this plant species now thrives in various regions worldwide, including Europe, Africa, and southwest Asia (Barnes et al., 2007).

Historically, *C*. *nobile* (L.), widely known as Roman chamomile, has been widely used as a remedy for various health conditions due to its rich composition of bioactive secondary metabolites. These include terpenes, flavonoids, and phenolic acids, which are responsible for its therapeutic properties, including antimicrobial and antioxidant activities (Aremu et al., 2018). Such bioactivities also suggest its potential for applications beyond healthcare, particularly in pest management.

Among these plants, *C. nobile* (L.) is renowned for its therapeutic properties, primarily attributed to its rich composition in secondary metabolites. Studies have demonstrated that *C. nobile* EO exhibits significant antioxidant activity by inhibiting lipid peroxidation and scavenging free radicals, effects largely linked to its phenolic content. Additionally, its antimicrobial activity has been shown to target a wide range of pathogens, including *Staphylococcus aureus*, *Escherichia coli*, and *Pseudomonas aeruginosa* (Bail et al., 2011). The bioactive compounds present in *C. nobile* EO, including flavonoids, terpenoids, and phenolic acids, are not only responsible for its therapeutic effects but may also contribute to its insecticidal efficacy, making this an area of great interest for sustainable pest management solutions.

However, despite its well-documented therapeutic properties, this study is the first to explore the insecticidal potential of *C. nobile* EO, particularly against *C. maculatus*. To our knowledge, no prior research has specifically assessed the efficacy of *C. nobile* EO as an insecticidal agent, making this work a pioneering contribution to the field. This originality lies not only in its focus on insecticidal activity but also in its application to *C. maculatus*, a major storage pest affecting agricultural productivity in our region.

This research aims to provide a comprehensive molecular and chemical profiling of *C. nobile* EO using GC-MS, evaluate its antioxidant capacity, and explore its antimicrobial and insecticidal properties. By focusing on its bioactivity against *C. maculatus*, the study highlights the potential of *C. nobile* EO as a natural alternative to synthetic insecticides and antimicrobials, particularly in the context of regional pest management challenges.

## 2 Materials and methods

### 2.1 Plant material and EO extraction

The flowers of *C. nobile* (L.) were collected on the morning of 1 April 2021, during the peak flowering period in the Tahar Souk region, situated approximately 50 km from Taounate, Morocco. The collection site is precisely located at 35°1′22″N and 4°8′27″W, at an altitude of 592 m. The region is characterized by a Mediterranean climate, with maximum temperatures reaching up to 40°C. After harvesting, the flowers were air-dried in the laboratory under controlled conditions, away from light and humidity, at room temperature. The CN-EO was extracted using a Clevenger apparatus in a 2 L flask, where 100 g of dried flowers were combined with 1 L of distilled water. Following the extraction, the EO was dehydrated using anhydrous sodium sulfate, filtered, and stored in flasks at 4°C for future use (El-Assri et al., 2021b; El Moussaoui et al., 2021).

### 2.2 Genomic DNA extraction, PCR, and sequencing

DNA extraction from randomly selected fresh *C. nobile* (L.) flowers of the same age followed the protocol described by Jawhari et al. (2023). The Rbcl (Ribulose-1,5 Bisphosphate Carboxylase) region was amplified by PCR with the universal primers rbcL a-f (5′-ATG​TCA​CCA​CAA​ACA​GAG​ACT​AAA​GC3′) and rbcL a-r (5′GTA​AAA​TCA​AGT​CCA​CCG​CG3′) (Parvathy et al., 2018). Amplification conditions were as follows: 35 cycles at 95°C for 4 min, 94°C for 30 s, 55°C for 1 min, 72°C for 1 min. The outcomes of the PCR were observed through electrophoresis on a 1.5% agarose gel.

Sequences were aligned and processed using ChromasPro sequence analysis software (version 2.1.10.1), and a BLAST search was conducted to identify homologous sequences in the GenBank database. The sequences were then deposited in GenBank. A phylogenetic tree was constructed from the obtained sequences using MEGA 5.0 software. *Artemisia giraldii* (OK128342) was chosen as the out-group. The maximum likelihood approach was employed to compute phylogenetic connections, and 1,000 bootstrap replications were conducted to evaluate the support for each branch in the resulting tree.

### 2.3 Oil of *C. nobile* (L.) identified using GC-MS

GC-MS The chemical composition of the essential oil (EO) was determined using a gas chromatograph (TRACE GC-ULTRA, S/N 20062969, Thermo-Fisher Scientific, Waltham, MA, United States) connected to a mass spectrometer (Quadrapole, PolarisQ, S/N 210729, Thermo Fisher Scientific, Waltham, MA, United States). The analysis employed an HP-5MS capillary column, measuring 50 m in length, with an internal diameter of 0.32 mm and a film thickness of 1.25 µm. The oven temperature was programmed to rise from 40°C to 280°C at a rate of 5°C per minute. The injector and detector (PolarisQ) temperatures were maintained at 250°C and 200°C, respectively. Electron-impact ionization (EI) at 70 eV was utilized. Helium was used as the carrier gas at a flow rate of 1 mL/min with a split ratio of 1:40. A 1 µL sample of EO was injected for analysis. The relative percentages of the constituents were calculated, and the compounds were identified by comparing their retention times with those in the NIST-MS Search Version 2.0 library (Atki et al., 2020; Mssillou et al., 2022).

### 2.4 Antioxidant activity *in vitro*

The DPPH (2,2-diphenyl-1-picrylhydrazyl) assay specifically evaluates the radical scavenging activity of the extract against DPPH radicals, which are widely used to measure antioxidant potential *in vitro*. The assay was performed following the methodology described by El Moussaoui et al. (2021). Briefly, a 0.004% (w/v) DPPH solution, equivalent to approximately 1.01 × 10⁻⁴ M, was prepared. Next, 100 µL of CN-EO extract, diluted to different concentrations in methanol, was added to 750 µL of the dissolved DPPH solution. A similar process was followed for butylated hydroxytoluene (BHT), a synthetic antioxidant, also diluted to different concentrations in methanol. Following a 30-min incubation period at ambient temperature, the spectrophotometer UV-Vis (JENWAY 85617) was employed to determine absorbance to 517 nm. DPPH % inhibition (PI %) was determined using the following equation:

$$\text{PI}\left(\%\right)=100\times \left(A0-A/A0\right)$$

• PI denotes the percentage inhibition.
• A0 represents the absorbance of the negative control (DPPH without the sample).
• A represents the absorbance of the sample with DPPH.

### 2.5 Assessing the antibacterial activity of CN-EO

The antibacterial effects of CN-EO were tested against four bacterial strains, namely *Proteus mirabilis* ATCC29906, *E. coli* K12, *Klebsiella pneumoniae* CIP A22, and *S. aureus* ATCC6633. The bacterial strains mentioned above were provided by the CHU Hassan II in Fez. The diameter of inhibition was determined using the disk diffusion technique. The strains were introduced into Petri dishes filled with Mueller-Hinton agar (MH) at a concentration of 10^6^–10^8^ CFU/mL (0.5 McFarland). Subsequently, 6 mm-diameter filter paper discs were saturated with 20 µL of CN-EO, and a positive control was conducted using streptomycin antibiotic (25 µg/disc). Incubation took place for 24 h at 37°C, and upon completion of the experiment, the diameter of growth inhibition zones was assessed (El Barnossi et al., 2020; Lafraxo et al., 2022).

### 2.6 Antifungal activity

We sought to measure the antifungal capacity of CN-EO against 4 species of fungi. These fungi are *Candida albicans* ATCC, *Aspergillus flavus*, *Fusarium oxysporum* MTCC9913, and *Aspergillus niger* MTCC282. To conduct the test, fungi were inoculated onto Petri dishes filled with malt agar extract medium. Subsequently, 6 mm Whatman paper discs were saturated with 20 µL of OE-MC. The tests were carried out with incubation periods of 7 days at 30°C for *A. niger*, *A. flavus* and *F. oxysporum*, and at 37°C for 24–48 h for *C. albicans*. Positive controls were also conducted using the antibiotic Fluconazole (15 mg/mL). After the incubation period, the inhibition diameter in mm was measured for *C. albicans*, while growth in mm was assessed for both negative and positive tests. This data was then used to calculate the percentage of inhibition for strains of filamentous fungi using the following formula (Moussaid et al., 2019):

$$\%Inhibition=\frac{\text{Negative}\;\text{test}-\text{Positive}\;\text{test}}{\text{Negative}\;\text{test}}\times 100$$

### 2.7 Establishing the minimum inhibitory concentration (MIC)

The MIC values of CN-EO compared to 4 fungal and 4 bacterial strains were determined using microdilution, following the protocol outlined by Sarker et al. (2007). In brief, a sterile 96-well microplate was employed. For bacterial and fungal strains, 50 µL of MH medium and malt extract (ME), respectively, were added to each well. CN-EO was diluted at a ratio of 1/10 (v/v) in 10% DMSO, and 100 µL of this solution was dispensed into the first column of the microplate. Subsequently, microbial strains (30 µL) were added following a 1:2 dilution series up to column 11. Plates were placed in incubators at 37°C or 30°C for 24 h, 48 h or 7 days, respectively for bacteria, *C. albicans* and filamentous fungi. Following the incubation period, 20 µL of a 0.2% solution of 2,3,5-triphenyl-tetrazolium-chloride was added to each well to facilitate the visualization of microbial growth (El Abdali et al., 2023). The MIC was characterized as the lowest concentration at which the absence of red coloration was noted, with the findings presented in mg/mL.

### 2.8 Insecticidal action against *C. maculatus*

#### 2.8.1 Fumigation test

The effectiveness of CN-EO vapours against *C. maculatus* was assessed through a fumigation experiment conducted in sealed 1 L containers. Whatman N°1 paper squares (3 × 3 cm) saturated with different concentrations of EO (4–20 μL/L of air) was attached to the inner surface of the container lids to avoid direct contact with the insects. Each container received ten pairs of *C. maculatus* aged 0–48 h, with repeated treatments and an untreated control group. Mortality rates were recorded daily for 5 days under controlled environmental conditions (temperature 27°C ± 1°C, relative humidity 70% ± 5%, and a light/dark photoperiod of 14:10). This process was continued until complete mortality of brushed insects was observed in all treated groups (Baghouz et al., 2022).

Total adult mortality calculated using Abbott’s equation (Abbott et al., 1925):

$$\text{Pv}=\frac{Pa-Pi}{100-Pi}\times 100$$

where: Pc: percentage mortality, Pa: indicates the mortality observed in the test and Pi: mortality observed in the negative control.

#### 2.8.2 Repellent effect of CN-EO

The repellence of CN-EO against *C. maculatus* adults was investigated using the preferential zone methodology on filter paper, as described by McDonald et al. (1970). Half-discs of Whatman N°1 paper (8 cm) were treated with different doses of CN-EO (4–20 μL/0.5 mL acetones) or with pure acetone (control). After drying, the halves were brought together and five pairs of adults were placed in the centre. Repellence was calculated 30 min later on the basis of insect distribution, using the formula of McDonald et al. (1970):

$$\mathrm{PR}\left(\%\right)=\frac{N\;–\;\text{NT}}{N+\text{NT}}\times 100$$

Repellence percentage (RP) is determined by comparing the number of *C. maculatus* adults found on the acetone-treated (control) side (N) to those on the essential oil-treated side (NT).

### 2.9 In-silico docking

In order to investigate how the *C. nobile* (L.) plant inhibits antioxidant, antibacterial, antifungal, and insecticidal functions, four specific proteins were selected as the key molecules to interact with β-Oplopenone, a primary compound found in this plant belonging to the Asteraceae family. To achieve this objective, an *in silico* molecular docking approach was conducted employing Autodock software. The targeted receptors, including the NADPH oxidase protein (2CDU.pdb), FimH lectin protein from *E. coli* K12 (4XO8.pdb), secreted aspartic protease from *C. albicans* (1ZAP.pdb), and insecticide-resistant crystal structure of an acetylcholinesterase mutant from the malaria vector *Anopheles gambiae* (6ARY.pdb), were obtained from the RCSB Protein Data Bank (PDB). Subsequently, these receptors were prepared according to the standard protocol, involving the removal of water molecules and co-crystallized ligands, along with the addition of Gasteiger charges (Benkhaira et al., 2023; Nouioura et al., 2024). Afterwards, the primed proteins were subjected to docking with the principal compound of the investigated plant utilizing Autodock software (Fadili et al., 2023). Finally, all resulting interactions were visualized using Discovery Studio software (Abechi et al., 2024).

### 2.10 Statistical analysis

Quantitative data are expressed as means ± standard deviation (SD) across three independent experiments. Statistical significance of differences between means was evaluated using two-way ANOVA, followed by Tukey’s multiple comparison test for *post hoc* analysis using GraphPad Prism 9 (El Barnossi and Iraqi, 2023).

## 3 Results and discussion

### 3.1 Molecular identification of *C. nobile* (L.)

PCR products amplified from the rbcL gene were approximately 600 bp in size. Sample A (*C. nobile* (L.)) exhibited strong amplification of the rbcL primer. BLAST analysis, conducted using the NCBI gene bank, confirmed that sequence A matched identically with *C. nobile* (L.), boasting an identity value of 99.29%. The sequence has been deposited in the GenBank database under the reference number OR838664, affirming its classification as *C. nobile* (L.).

**FIGURE 1:**
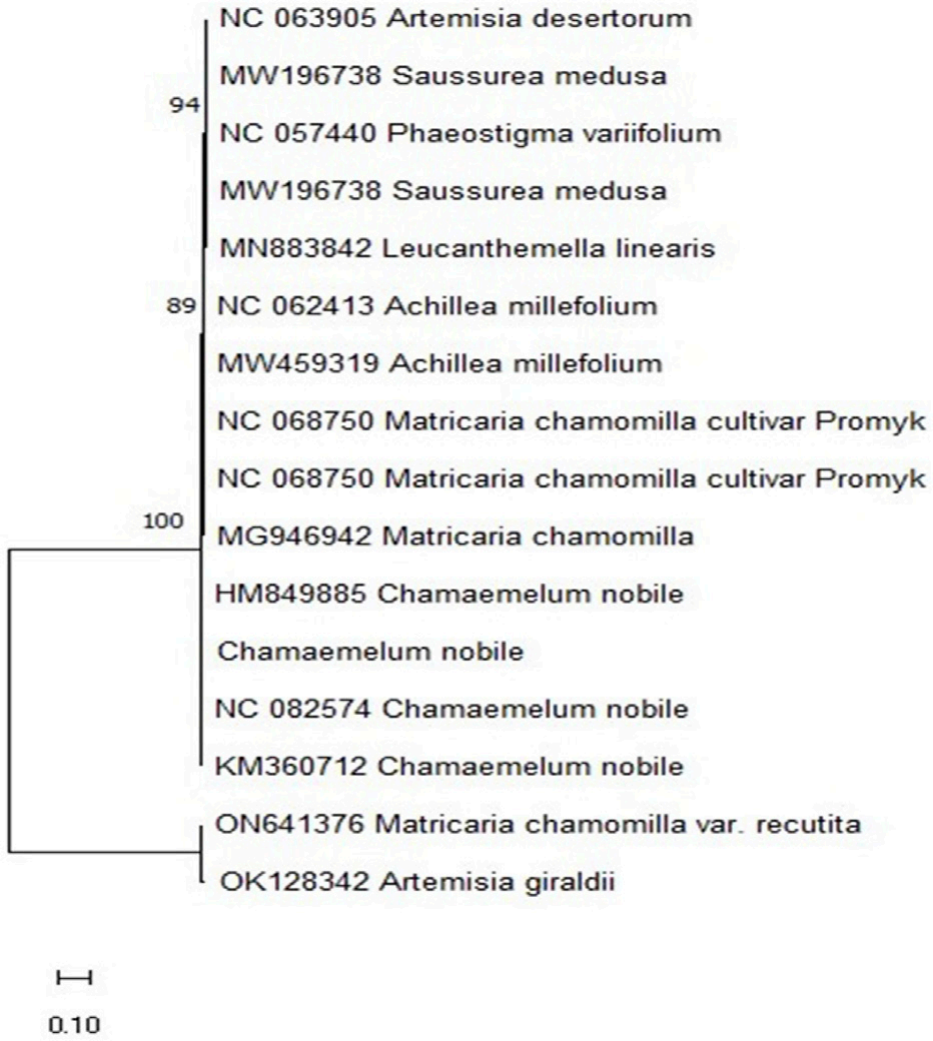
Phylogenetic analysis of *C. nobile* (L.) OR838664 samples.

The Neighbor Join method, employed for phylogenetic analysis, underscores the significance of the rbcL gene in species identification and classification. This genetic tool leverages similarities among species to construct phylogenetic trees, facilitating the understanding of inter-species relationships (Sundari et al., 2019). Phylogenetic trees elucidate species relationships via genetic analysis. Notably, the plant sample in question was clustered within the same clade as C. nobile HM849885 (Figure 1), underscoring its substantial genetic affinity.

### 3.2 The yield and essential oils

**FIGURE 2:**
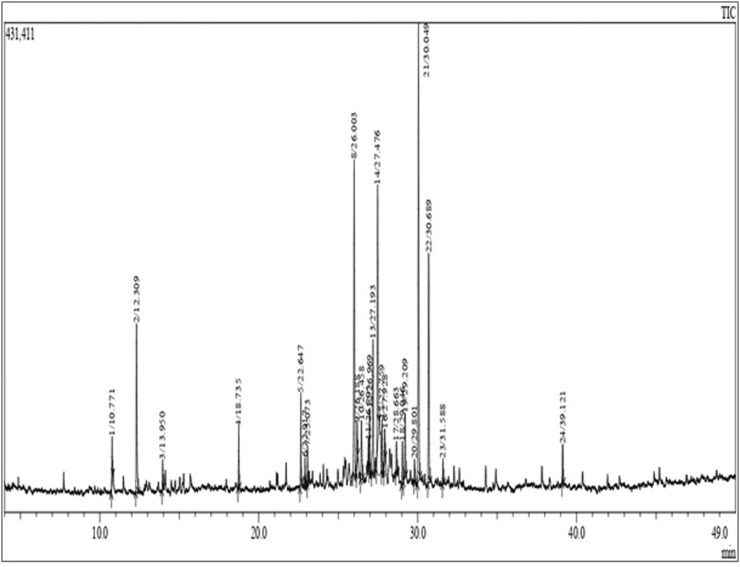
GC-MS chromatogram of *C. nobile* (L.) EO.

*C. nobile* (L.) flowers, suitable for essential oil extraction through hydro-distillation, typically yield around 0.4%, a figure consistent with the findings of Abdoul-Latif et al. (2021) at 0.44%. However, this yield falls below those documented by Ailli et al. (2023) and Aremu et al. (2018), who reported yields of 1.3% and 1.2%, respectively. Analysis of CN-EO revealed a diverse composition of 24 distinct compounds, together accounting for 99.56% of the total composition (Figure 2 and Supplementary Table S1). Sesquiterpenes dominate, constituting 81.20%, followed by monoterpenes at 15.41%. Among the predominant compounds, β-Oplopenone is notable with a concentration of 18.66%. Additionally, Spathulenol (14.90%), Himachalene (12.47%), Ionone (9.52%), Phenyl-tert-butanol (7.20%), β-Humulene (6.34%), α-Copaene (3.15%), β-Farnesene (3.05%), and α-Terpinene (3.04%) are identified. The remaining compounds are present in concentrations below 3%.

Numerous studies have delved into the chemical composition of Roman chamomile. In the Khenifra region of Morocco, Abdoul-Latif et al. (2021) identified verbenone as the primary constituent (33.74%), followed by pulegone (26.45%). In France, Tadrent et al. (2016) reported that Roman chamomile EO is abundant in esters, with isobutylangelate (35.9%–38.5%), isoamylangelate (18%), isobutylisobutanoate (6%), and 2-methylbutyl angelate (0.1%–20.3%) being major components. Studies in Iran revealed distinct compositions of CN-EO: Dezfooli et al. (2012) identified alpha-bisabolol oxide A (58.8%) and isopropyl hexadecanoate (15%), while Farhoudi and Lee (2017) highlighted chamazulene (31.12%), beta-pinene (10.11%) and alpha-bisabolol (7.32%). Research in South Africa by Aremu et al. (2018) emphasized alpha-bisabolol (50%) as the predominant constituent, alongside farnesene (5.35%) and spathulenol (2.56%). Farhoudi (2013) identified chamazulene (27.80%), 1,8-cineole (7.51%), and alpha-bisabolol (5.76%) as primary compounds. Additionally, Radulovi et al. (2009) noted isobutyl isobutanoate (4.4%), 2-methylbutyl isobutanoate (4.3%), isobutyl angelica (24.5%), 2-butenyl angelica (7.3%), and 2-methylbutyl angelica (17.4%) in the same plant source.

### 3.3 Scavenging of the free radical DPPH^•^

**FIGURE 3:**
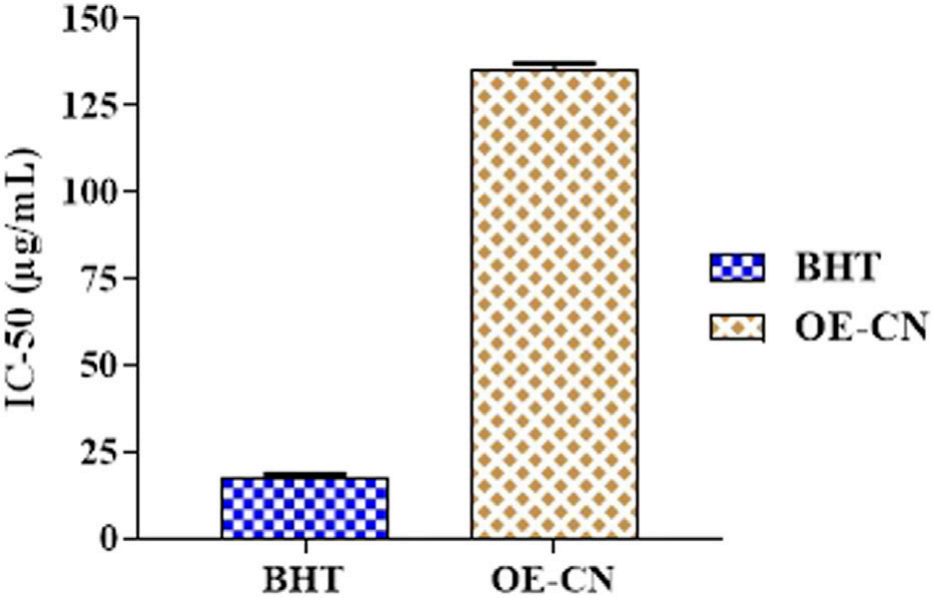
DPPH test for antioxidant activity of EO and BHT (µg/mL).

In this research, we explored the antioxidant potential of CN-EO using the DPPH assay, a method widely recognized for assessing the ability of substances to neutralize free radicals. Our results demonstrate that CN-EO exhibits dose-dependent antioxidant activity. The highest inhibition of DPPH, reaching 61%, was observed at 350 μg/mL. The IC_50_ value, which represents the dose required to inhibit 50% of DPPH radicals, was calculated to be 135.8 ± 1.03 μg/mL (Figure 3). This value is significantly higher than that of BHT (17.0 ± 0.76 μg/mL), a standard synthetic antioxidant, indicating that CN-EO has a lower antioxidant capacity compared to BHT. However, the moderate antioxidant potential of CN-EO can be attributed to the presence of various antioxidant molecules in its composition, such as β-Oplopenone, Spathulenol, and Himachalene.

Our findings are consistent with those reported by Farhoudi (2013), which demonstrated that *C. nobile* oil exhibits antioxidant properties with an IC50 value of 195.8 μg/mL. Conversely, the research by Ailli et al. (2023) found a lower antioxidant activity, with inhibition percentages reaching 47.98%. Additionally, Sharifzadeh et al. (2015) reported even lower values, with an IC_50_ of 602.73 ± 4.8 μg/mL. These comparisons validate the significant antioxidant capacity of CN-EO, which surpasses that of other studies, likely due to its rich composition of antioxidant compounds.

### 3.4 Antibacterial evaluation

The anti-bacterial potential of CN-EO was assessed against four bacterial strains, encompassing one Gram-positive and three Gram-negative bacteria. The study revealed significant activity against all four strains, demonstrating zones of inhibition ranging from 10.33 ± 0.58 mm to 20.67 ± 0.58 mm (Supplementary Table S2), using the disk diffusion technique. The largest inhibition zone was observed for *S. aureus* (20.67 ± 0.58 mm), followed by *E. coli* (18.69 ± 1.53 mm) and *P. mirabilis* (15.5 ± 1.03 mm), with the smallest inhibition zone for *K. pneumoniae* (10.33 ± 0.58 mm). Additionally, the study determined MICs ranging from 2.53 ± 0.00 to 10.12 ± 0.00 µg/mL, with the highest MIC for *P. mirabilis* at 10.12 ± 0.00 µg/mL and the lowest for *S. aureus* at 2.53 ± 0.00 µg/mL.

CN-EO harvested in Khenifra, Morocco, demonstrated significant antibacterial activity against various bacterial strains. The oils exhibited strong inhibition against *P. aeruginosa* (16.1 ± 1.44 mm) with an MIC of 52.61 μL/mL and *S. epidermidis* (15.65 ± 0.34 mm) with an MIC of 26.67 μL/mL. Smaller inhibition diameters were recorded against *E. coli* (14.45 ± 0.25 mm) and *Listeria innocua* (12.6 ± 0.72 mm) (Abdoul-Latif et al., 2021). These findings align with research conducted worldwide. For example, Dezfooli et al. (2012) in Iran showed the antibacterial efficacy of CN-EO against *Pseudomonas tolaasii*, reporting a zone of inhibition of 3.5 mm. Similarly, Chao et al. (2011) in the United States observed moderate activity against *Bacillus cereus* (ATCC 14579), *E. coli* (ATCC25922), *Micrococcus luteus* (ATCC4698), *S. aureus* (ATCC259231) and *P. aeruginosa* (ATCC27853), with inhibition zones ranging from 1 mm to 9 mm. Furthermore, NC-EO flowers grown in Provence, France, demonstrated potent antimicrobial effectiveness against various bacterial strains, including *K. pneumonia, S. aureus*, *Escherichia faecalis*, *P. aeruginosa*, *P. vulgaris*, and *Salmonella*. The inhibition zones range from 9 to 20 mm (Bail et al., 2011). *C. nobile* (L.) from Egypt also demonstrated activity against Gram-negative bacteria (Abobakr et al., 2016). In a research conducted by Saderi et al. (2005) showed significant inhibitory activity of CN-EO against *Porphyromonas gingivalis*, with a mean zone of inhibition of 20.5 ± 0.5 mm. Conversely, Kloucek et al. (2012) indicated that CN-EO did not exhibit antibacterial effects against *Salmonella enteritidis*, *P. aeruginosa* and *S. aureus*. Additionally, Ailli et al. (2023) tested CN-EO against eight bacterial strains at various concentrations, revealing significant antibacterial activity across all tested bacteria. These results are by previous studies suggesting that Gram-positive bacteria tend to be more sensitive to antibacterial agents than Gram-negative bacteria (Barbosa et al., 2015; Kazemi, 2015). This consistency highlights the potential of CN-EO as a potent antibacterial agent, particularly effective against Gram-positive bacteria.

### 3.5 Antifungal activity

Supplementary Table S3 presents the inhibition percentages and MIC values of CN-EO against four fungal strains, demonstrating its capability to reduce fungal growth to varying extents. The greatest inhibition zone was noted for *A. flavus* (40.42 ± 2.82 mm), succeeded by *C. albicans* (38.17 ± 0.76 mm). Conversely, CN-EO exhibited lower inhibition diameters against *A. niger* and *F. oxysporum*, with inhibition percentages of 23.47 ± 0.7 and 22.45 ± 0.40, respectively. The MIC values of CN-EO spanned from 0.0025 ± 0.00 to 0.020 ± 0.00 mg/mL, showcasing diverse antifungal efficacy against various fungal strains.

Several studies have confirmed the antifungal properties of CN-EO EO (Móricz et al., 2012; Abobakr et al., 2016). Based on the study by Ghalem et al. (2020), CN-EO demonstrated an antifungal index of 81.48% at 2.5 µL/mL against *A. alternata*. The effectiveness of CN-EO’s antifungal properties can vary depending on the strain and dose used. For instance, a study by Al-snafi, (2016) found that CN-EO suppressed the growth of various *Aspergillus fumigatus*and *A. parasiticus*. However, CN-EO showed no activity against *Cryptococcus neoformans*, *C. albicans*, *Histoplasma capsulatum*, and *A. niger*. In research conducted by Sharifzadeh et al. (2015), CN-EO displayed moderate to low inhibitory activity against fungal pathogens responsible for foodborne illnesses, with MIC values varying between 3,000 and 5,000 µg/mL. Similarly, A prior study conducted by Magro et al. (2006) validated the inhibitory impact of CN-EO on species of *Aspergillus* and *Penicillium*. In contrast, a report by Kloucek et al. (2012) indicated that CN-EO had no antifungal effect against *A. alternata*, *A. niger*, and *P. digitatum*.

These studies collectively highlight the antifungal potential of CN-EO, although its efficacy can vary significantly based on the specific fungal strain and concentration used.

### 3.6 Assessment of insecticidal activity

#### 3.6.1 Fumigation test

**FIGURE 4:**
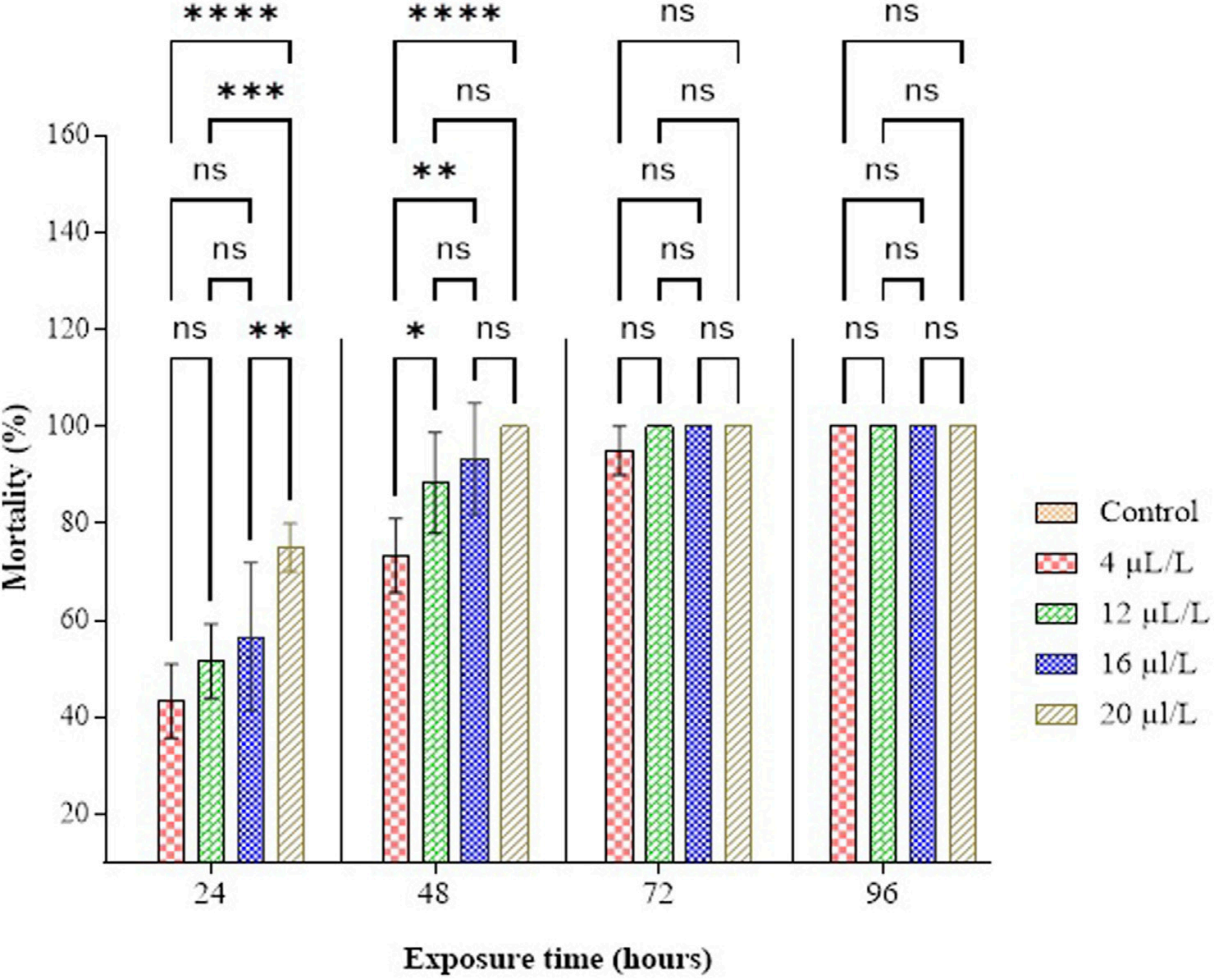
Percentage mortality of *C. maculatus* adults as a function of concentration and duration of exposure to CN-EO. Means (±SD, n = 3) marked with a star indicate a significant difference according to two-way ANOVA and Tukey’s multiple comparison tests. The symbols represent p-values as follows: ns: not significant (p > 0.05), *: p ≤ 0.05, **: p ≤ 0.01, ***: p ≤ 0.001, ****: p ≤ 0.0001.

The findings from the study, illustrated in Figure 4 and Supplementary Table S4, reveal that the mortality rate of adult *C. maculatus* increases with both the administered dose and the duration of exposure. The statistical examination of the data unveiled a noteworthy correlation among the adult mortality rate, administered dose, and duration of exposure (F = 511.44; df = 4, 40; P < 0.0001; F = 109.88; df = 3, 40; P < 0.0001). Notably, after 48 h of exposure, CN-EO exhibited high toxicity at 20 and 16 µL/L of air, with total insect mortality achieved within 48 h at 16 µL/L. Additionally, total mortality was observed after 96 h of exposure at doses of 4 and 12 µL/L of air. The median lethal concentration (LC_50_) of CN-EO was determined to be 1.90 µL/L of air after 48 h, with a 95% confidence interval of 0.015–4.023). It is important to note that no mortality was observed in the untreated control group (0 ± 0 across all exposure durations), confirming the absence of external factors influencing the observed effects. These findings indicate that the compounds in CN-EO are highly toxic to adult *C. maculatus.*

Herbal medicines are recognized as effective bio-insecticides for controlling various insect pests (Devrnja et al., 2022). Various studies have shown the substantial advantages of Eos in decreasing insect pest populations, including *C. maculatus*. EOs and their components, notably monoterpenes and sesquiterpenes, are highly volatile and exhibit insecticidal properties that interfere with insect growth at different developmental stages (Zekri et al., 2013; El Moussaoui et al., 2022). Our findings support the effectiveness of CN-EO against *C. maculatus.* Previous studies have shown that CN-EO can result in 100% mortality among *termites* after 48 h of treatment with an air concentration of 10 mg/L (Seo et al., 2014). Similarly, Kim et al. (2013) observed total mortality in *Metcalfa pruinosa* following a 24-h treatment with a dose of 1 mg/L of *C. nobile*. Al-snafi, (2016) also demonstrated notable efficacy of this EO against *Trialeurodes vaporariorum*, with effective concentrations at 0.0047 and 0.009 µg/mL. The LC50 values obtained for this EO are noteworthy, with an LC50 of 0.96 mg/mL against the *tick Hyalomma scupense* (Alimi et al., 2024). Furthermore, exposure to CN-EO resulted in an LC_50_ of 8.57 µg/fly for *Aphis suspensa*, while the LC_99_ value was 22.38 µg/µL (Ali et al., 2023).

#### 3.6.2 Repellency test

Essential oils impact insects through various mechanisms, including disrupting growth, moulting, and development, inducing sterility, altering behaviour, damaging the digestive tract membrane, causing metabolic disorders, inducing neuromuscular toxicity, and causing nonspecific multisite inhibitions (Regnault-Roger et al., 2012; Isman, 2019). For centuries, people have used aromatic plants in phytotherapy as insect repellents (Pavela, 2016). Numerous investigations have evaluated the efficacy of chamomile species in deterring agricultural pests (Mousavi et al., 2020).

The repellent properties of CN-EO against *C. maculatus* were assessed using the preferential surface area method on filter paper, outlined in Supplementary Table S5. The findings indicated that the repellent effectiveness of CN-EO was dose-dependent. The findings indicated that the repellent efficacy of CN-EO varied with dosage. At the minimum concentration of 4 μL/cm^2^, a repellency of 45% ± 10% was observed, while concentrations of 12 and 16 μL/cm^2^ resulted in repellencies of 55% ± 10% and 65% ± 10%, respectively. The highest concentration of 20 μL/cm^2^ exhibited a repellency of 75% ± 10% against adult *C. maculatus*. Repellency percentages obtained using the Mc Donald et al. (1970) method indicated that CN-EO was effectively repellent, with rates of 60%.

These findings align with the study by Abd El-Aziz and El-Sayed, (2009), which demonstrated the efficacy of CN-EO against *Tribolium confusum* adults and larvae within 24 h. Their results showed similar repellent effects on both adults and larvae, with repellency rates of 73.88% ± 2.91% for adults and 62.90% ± 6.34% for larvae. Another investigation by Alimi et al. (2024) found that CN-EO provided a powerful repellent effect, achieving a rate of 95.98% at a 4 mg/mL against the *H. suspense tick*. The observed repellent effect could stem from the synergistic interaction among the constituents of the oil or from the notable biological activity of minor compounds found within the oils (Murugesan et al., 2021).

### 3.7 Molecular docking

**FIGURE 5:**
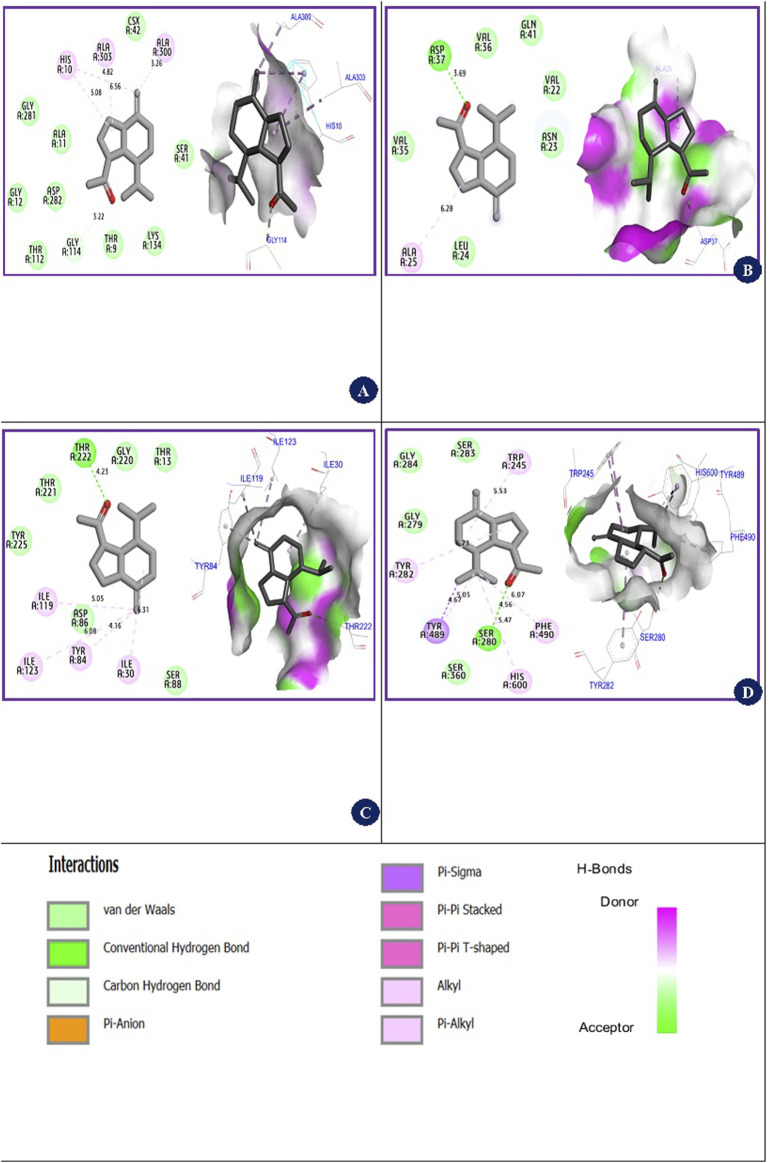
2D/3D views of inhibition mechanisms of β-Oplopenone ligand in complex with 2CDU.pdb, 4XO8.pdb, 1ZAP.pdb, and 6ARY.pdb proteins with binding energies of −7.16 kcal/mol, −5.24 kcal/mol, −5.48 kcal/mol, and −8.16 kcal/mol, respectively. **(A)** (β-Oplopenone ligand-2CDU.pdb protein) complex. **(B)** (β-Oplopenone ligand-4XO8.pdb protein) complex. **(C)** (β-Oplopenone ligand-1ZAP.pdb protein) complex. **(D)** (β-Oplopenone ligand-6ARY.pdb protein) complex.

The molecular docking simulations depicted in Figure 5 reveal the interactions between the β-Oplopenone compound and various proteins. For the NADPH oxidase protein, β-Oplopenone exhibited a binding energy of −7.16 kcal/mol, forming Alkyl and Pi-Alkyl bonds with amino acid residues Ala300, Ala303, and His10 in the A chain. Similarly, the compound interacted with the antibacterial protein coded as 4XO8.pdb, showing a binding energy of −5.24 kcal/mol. This interaction involved one hydrogen bond with the Asp37 amino acid residue and multiple alkyl bonds with the Ala25 residue in the A chain.

Furthermore, β-Oplopenone was docked with the antifungal protein from *C. albicans* (1ZAP.pdb), displaying a binding energy of −5.48 kcal/mol. This interaction resulted in one hydrogen bond with the Thr222 residue, along with four Alkyl bonds involving Ile119, Ile123, Ile30, and Tyr84 residues in the A chain. The ligand underwent docking with an acetylcholinesterase mutant resistant to insecticides, derived from the malaria vector *A. gambiae* (6ARY.pdb), yielding a binding energy of −8.16 kcal/mol. This interaction featured diverse intermolecular interactions with residues such as Ser280, Tyr280, Phe490, Tyr489, Trp245, and His600.

The binding energy observed during molecular docking simulations reflects the strength of the interaction between β-Oplopenone and specific proteins, which is indicative of the compound’s potential biological activity. For instance:

The interaction with NADPH oxidase (−7.16 kcal/mol) suggests that β-Oplopenone may inhibit reactive oxygen species (ROS) production by stabilizing the protein’s active site. This mechanism aligns with its potential antioxidant activity, as NADPH oxidase is a key enzyme responsible for ROS generation in oxidative stress conditions. The interaction with the antifungal protein from *C. albicans* (−5.48 kcal/mol) demonstrates hydrogen and alkyl bonding with active site residues such as Thr222, Ile119, and Ile123. These interactions could disrupt the protein’s normal function, impairing fungal cell growth or survival, which aligns with β-Oplopenone’s antifungal activity. The high binding affinity with the acetylcholinesterase mutant (−8.16 kcal/mol) indicates potential insecticidal activity by blocking the active site and preventing substrate binding, which would impair neural transmission in the malaria vector *A. gambiae*. The antibacterial protein (4XO8.pdb) interaction (−5.24 kcal/mol) through hydrogen and alkyl bonds may inhibit key enzymatic functions necessary for bacterial survival.

**FIGURE 6:**
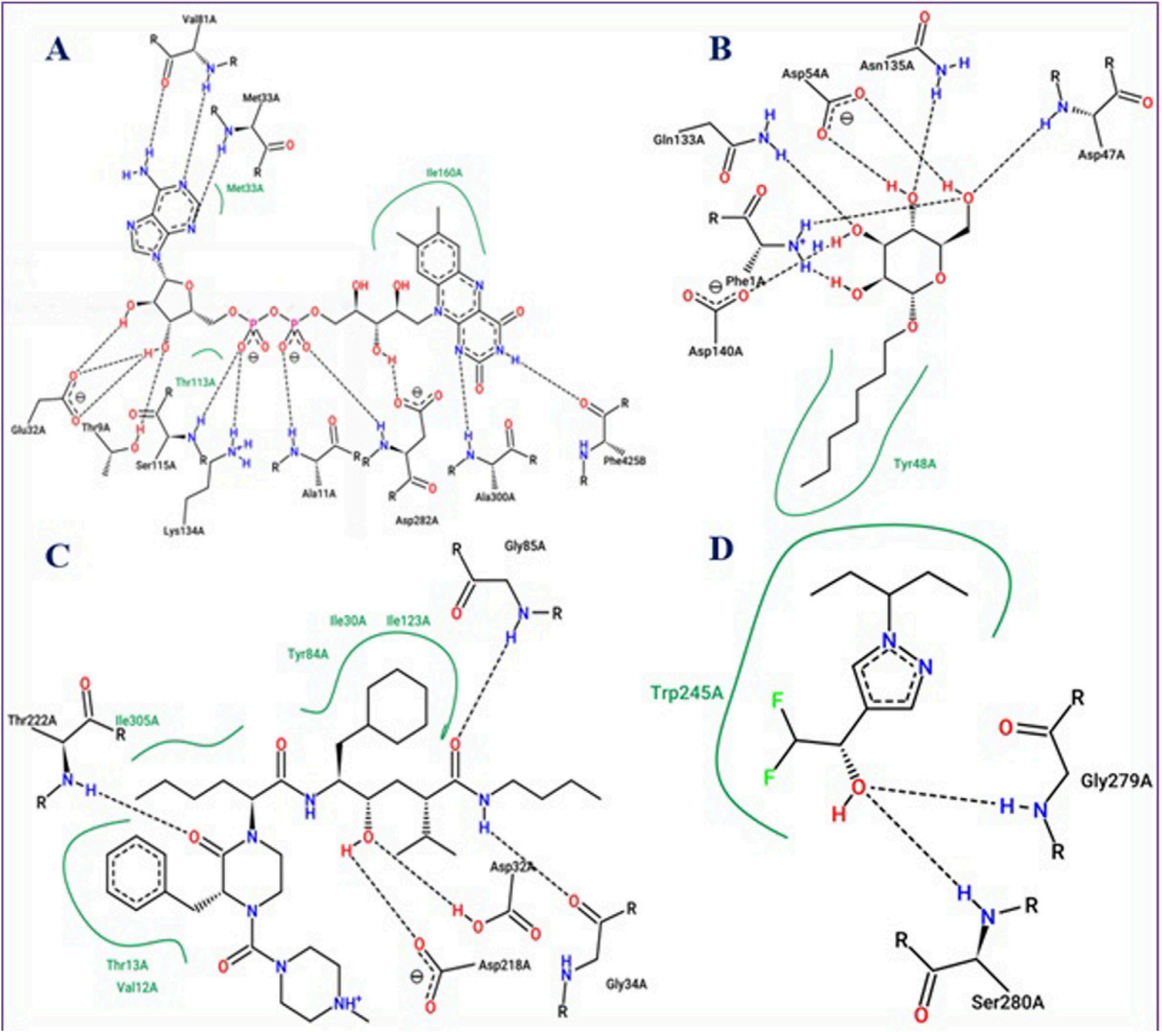
Active sites of NADPH oxidase protein (2CDU.pdb) **(A)**, FimH lectin protein from *Escherichia coli* K12 (4XOB.pdb) **(B)**, secreted aspartic protease from Candida albicans (1ZAP.pdb) **(C)**, and Crystal structure of an insecticide-resistant acetylcholinesterase mutant from the malaria vector Anopheles gambiae (6ARY.pdb) **(D)**, with their co-crystallized ligands: namely flavin-adenine dinucleotide, Heptyl alpha-D-mannopyranoside, N-ethyl-N-(4-methylpiperazin-1-ylcarbonyl)-D-phenylalaninamide, L-norepinephrine, and Difluoromethyl ketone inhibitor, respectively.

To evaluate the performance of these molecular docking processes, the intermolecular interactions of β-Oplopenone were compared to those of co-crystallized ligands for each targeted receptor. The results showed that β-Oplopenone docked with the lowest possible binding energies (in kcal/mol), interacting with Ala300 at the active site of the 2CDU.pdb protein, Thr222 at the active site of the 1ZAP.pdb protein, and Ser280 at the active site of the 6ARY.pdb protein, as depicted in Figure 6.

These molecular docking results suggest that β-Oplopenone’s pharmaceutical properties, including antioxidant, antifungal, and antibacterial activities, are closely tied to its ability to form strong, specific interactions with target proteins, thereby disrupting their normal biological functions.

## 4 Conclusion

In a recent study, we analyzed the chemical composition of CN-EO and conducted molecular characterization of *C. nobile*. The findings revealed that CN-EO comprises a diverse array of sesquiterpene and monoterpene compounds with multifaceted biological activities, these activities include antioxidant, antibacterial, antifungal and insecticidal properties. These properties suggest that CN-EO holds potential for various biological applications, particularly in the development of novel pharmaceutical products and as a natural preservative for fresh food in the food industry, offering an alternative to chemical preservatives. While this investigation establishes a robust groundwork for potential applications of CN-EO, further research is imperative to fully harness its advantages. *In vivo* and clinical studies are crucial for validating its pharmacological effects and ensuring its safety and efficacy. Additionally, thorough toxicity evaluations are essential to ascertain appropriate usage levels and mitigate any potential adverse effects.

## Data Availability

The datasets presented in this study can be found in online repositories. The names of the repository/repositories and accession number(s) can be found in the article/Supplementary Material.
