# Supplementary material for: Molecular characterization, chemical profile and biological properties of essential oils from Chamaemelum nobile (L.) flowers of Morocco: in vitro and in silico studies
Source: Front Chem. 2025 Feb 4;13:1539872. doi: 10.3389/fchem.2025.1539872 (PMC11832495; doi:10.3389/fchem.2025.1539872)
Supplement: Supplementary file 1 [file Table1.docx]

Supplementary Material

# Supplementary Tables

**Table 1S**

Phytochemical composition of CN-EO determined via GC/MS

| Peak | Retention time | Compound | Retention index | Chemical formula | Terpene class | Area (%) |
| --- | --- | --- | --- | --- | --- | --- |
| 1 | 10.771 | Santolina triene | 908 | C10H16 | MNT | 1.48 |
| 2 | 12.309 | Phenyl-tert-butanol | 1158 | C10H14O | MNT | 7.20 |
| 3 | 13.950 | Nerolidol | 1532 | C15H26O | MNQ | 0.98 |
| 4 | 18.735 | Limonen-10-ol | 1289 | C10H16O | MNT | 1.87 |
| 5 | 22.647 | β -Farnesene | 1442 | C15H24 | MNQ | 3.05 |
| 6 | 22.917 | Terpinolene | 1088 | C10H16 | MNT | 0.72 |
| 7 | 23.073 | Borneol | 1169 | C10H18O | MNT | 1.10 |
| 8 | 26.003 | Spathulenol | 1578 | C15H24O | MNQ | 14.90 |
| 9 | 26.188 | Caryophyllene oxide | 1583 | C15H24O | MNQ | 1.78 |
| 10 | 26.458 | α-terpinene | 1017 | C10H16 | MNT | 3.04 |
| 11 | 26.895 | Isobornyl n-butanoate | 1475 | C14H24O | O | 1.07 |
| 12 | 26.969 | Longiborneol | 1599 | C15H26O | MNQ | 2.74 |
| 13 | 27.193 | β-Humulene | 1438 | C15H24 | MNQ | 6.34 |
| 14 | 27.476 | Himachalene | 1482 | C15H24 | MNQ | 12.47 |
| 15 | 27.759 | Caryophylla-4(12),8(13)-dien-5a-ol | 1640 | C15H24O | MNQ | 2.14 |
| 16 | 27.928 | Muurolol | 1642 | C15H26O | MNQ | 1.45 |
| 17 | 28.663 | Aromadendrene | 1441 | C15H24 | MNQ | 1.24 |
| 18 | 29.046 | Caryophyllene | 1408 | C15H24 | MNQ | 1.98 |
| 19 | 29.209 | α-Copaene | 1376 | C15H24 | MNQ | 3.15 |
| 20 | 29.801 | Santalol | 1675 | C15H24O | MNQ | 0.80 |
| 21 | 30.049 | β-Oplopenone | 1607 | C15H24O | MNQ | 18.66 |
| 22 | 30.689 | Ionone | 1567 | C15H24O | MNQ | 9.52 |
| 23 | 31.588 | octadecane | 1800 | C18H38 | O | 0.86 |
| 24 | 39.121 | Octadecanol | 2077 | C18H38O | O | 1.43 |
|  |  | **Terpene class** | | | |  |
|  |  | Monoterpenes (MNT) | | | | 15.41 |
|  |  | Sesquiterpenes (MNQ) | | | | 81.20 |
|  |  | Diterpenes (MND) | | | | 0.0 |
|  |  | Triterpenes (MTT) | | | | 0.0 |
|  |  | Other (O) | | | | 3.36 |
|  |  | Total | | | | 99.97 |

**Table 2S**

CN-EO antibacterial activity (mm) and MIC (µg/mL) against the different bacterial strains tested.

|  | | CN-EO | Streptomycin |
| --- | --- | --- | --- |
| *E. coli* ATCC29213 | Diameter of Inhibition Zone | 18.69±1.53 **^a^** | 20.90 ± 0.36 **^a^** |
|  | MIC | 5.06 ± 0.00 **^a^** | 1.56 ± 0.00 **^b^** |
| *S. aureus* ATCC6633 | Diameter of Inhibition Zone | 20.67±0.58 **^a^** | 22.50 ± 0.20 **^a^** |
|  | MIC | 2.53 ± 0.00 **^a^** | 1.56 ± 0.00 **^a^** |
| *K. pneumoniae* CIP A22 | Diameter of Inhibition Zone | 10.33±0.58 **^a^** | 17.60 ± 0.36 **^b^** |
|  | MIC | 5.62 ± 0.00 **^a^** | 3.12 ± 0.00 **^a^** |
| *P. mirabilis* ATCC29906 | Diameter of Inhibition Zone | 15.5 ± 1.03 **^a^** | 16.73 ± 0.25 **^a^** |
|  | MIC | 10.12 ± 0.00 **^a^** | 3.12 ± 0.00 **^b^** |

Means (± SD, n=3) with dissimilar letters within the same row signify a significant distinction (ANOVA II, Tukey tests at p < 0.05).

**Table 3S**

Antifungal activity (inhibition %) and MIC (µg/mL) of CN-EO against fungal strains tested.

|  | | CN-EO | Fluconazole |
| --- | --- | --- | --- |
| *C. albicans* ATCC10231 | Diameter of Inhibition Zone (mm) | 38.17±0.76 **^a^** | 41.33 ± 1.15 **^a^** |
|  | MIC | 0.0025 ± 0.00 **^a^** | 0.012 ± 0.00 **^b^** |
| *A. niger* MTCC282 | Percentage of inhibition (%) | 23.47 ± 0.70 **^a^** | 47.67 ± 1.53 **^b^** |
|  | MIC | 0.020 ± 0.00 **^a^** | 0.0062 ± 0.00 **^b^** |
| *A. flavus* MTCC9606 | Percentage of inhibition (%) | 40.42 ± 2.82 **^a^** | 43.67 ± 1.53 **^a^** |
|  | MIC | 0.010 ± 0.00 **^a^** | 0.012 ± 0.00 **^a^** |
| *F. oxysporum* MTCC9913 | Percentage of inhibition (%) | 22.45 ± 0.40 **^a^** | 59.17 ± 0.76 **^b^** |
|  | MIC | 0.020 ± 0.00 **^a^** | 0.0062 ± 0.00 **^b^** |

Means (± SD, n=3) with dissimilar letters within the same row signify a significant distinction (ANOVA II, Tukey tests at p < 0.05).

**Table 4S**

LC50 and χ2 of CN-EO against *C. maculatus* adults.

|  | Days | LC_50_ (μl/L) | 95% CI | LC_90_ (μl/L) | 95%CI | *Df* | *χ2* |
| --- | --- | --- | --- | --- | --- | --- | --- |
| CN-EO | 24  48  72  96 | 7.28  1.90  0.45  --- | ---  0.015-4.023  ---  --- | 175.63  10.13  2.55  --- | ---  5.63-31.60  ---  --- | 2  2  2  --- | 1.43  1.26  0,37  --- |

**Table 5S**

Repulsion of *C. maculatus* by CN-EO.

| Dose (µL/cm^2^) | RI (Mean ± SD) | Repellency Class | The Average Rate of Repulsively (%) |
| --- | --- | --- | --- |
| CN-EO | | | |
| 4 | 45 ± 10 | Moderately repellent | 60  (Repellent) |
| 13 | 55 ± 10 | Moderately repellent |  |
| 16 | 65 ± 10 | Repellent |  |
| 20 | 75 ± 10 | Repellent |  |
